# Fatal case of disseminated toxoplasmosis following allogeneic stem cell transplantation in Singapore – a case report and review of literature

**DOI:** 10.1016/j.lrr.2025.100545

**Published:** 2025-09-10

**Authors:** Jinghao Nicholas Ngiam, Thuan Tong Tan, Ban Hock Tan, Wenlu Hou, Aloysius Yew Leng Ho, Jeffrey Kim Siang Quek, Yeh Ching Linn, Francesca Lorraine Wei Inng Lim, Hein Than, Shimin Jasmine Chung

**Affiliations:** a Department of Infectious Diseases, Singapore General Hospital, Singapore; b Division of Infectious Diseases, Department of Medicine, National University Health System, Singapore; c SingHealth Duke-NUS Transplant Centre, Singapore; d Department of Neuroradiology, Singapore General Hospital, Singapore; e Department of Hematology, Singapore General Hospital Singapore

**Keywords:** Toxoplasmosis, Hematopoietic stem cell transplant, HSCT, Allogeneic stem cell transplant, Case report

## Abstract

Toxoplasmosis is a rare but potentially fatal complication post-allogeneic hematopoietic stem cell transplantation (HSCT), often due to latent reactivation. In Singapore, low seroprevalence limits routine screening and prophylaxis. We report the first reported case of disseminated toxoplasmosis following HSCT in Singapore. A 58-year-old woman with fever and altered mental status two months post-transplant. She had pancytopenia, acute kidney injury, and pneumonitis, with non-specific brain MRI findings. Toxoplasma polymerase chain reaction from serum, bone marrow, and cerebrospinal fluid was positive. Despite treatment with trimethoprim-sulfamethoxazole, pyrimethamine, and sulfadiazine, she developed seizures, intracranial haemorrhage, and nosocomial infections, ultimately succumbing one month later. This case potentially highlights the consideration of routine pre-transplant Toxoplasma screening and prevention strategies, even in regions with low seroprevalence.

## 1 Case presentation

A 58-year-old lady from Indonesia with a history of myelofibrosis that had transformed into acute myeloid leukaemia (AML) received haematopoietic allogeneic stem cell transplantation (HSCT) from a HLA-matched sibling donor. She had no other medical co-morbidities. Two months post-transplant, she presented with unrelenting fever and altered mental status for five days. She was on acyclovir prophylaxis and due to issues of cytopenias, she received intravenous pentamidine for *Pneumocystis jirovecii* pneumonia (PJP) prophylaxis, in place of trimethoprim-sulfamethoxazole (TMP-SMX) [1]. Due to low prevalence of disease in our region, Toxoplasma serology is not routine, and her toxoplasma serostatus was unknown.

**Fig. 1:**
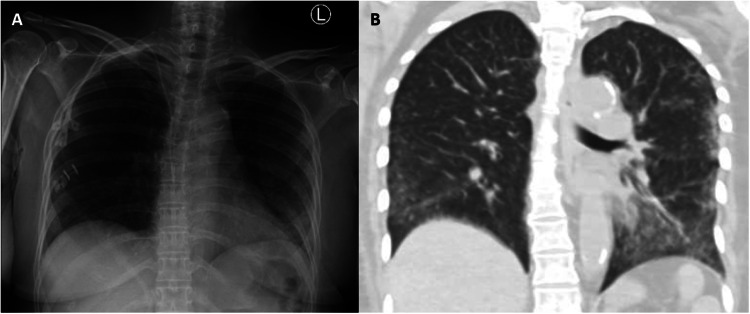
Chest radiology of a patient with disseminated toxoplasmosis. (A) Chest radiograph with diffuse bilateral peripheral infiltrates and (B) Computed tomography of the chest showing diffuse bilateral ground glass opacities consistent with bilateral pneumonitis.

At presentation, she was febrile (temperature 38.9 °C), tachycardic (heart rate 117 beats per minute), saturating at 96 % on room air and had a Glasgow coma scale of 9 (opened her eyes to voice, no verbal response and was localising pain). She was pancytopenic (haemoglobin 10.9 g/dL, white blood count 1.07 × 10^9^/L, with an absolute neutrophil count of 0.54 × 10^9^/L, and platelet count of 36 × 10^9^/L), and had acute on chronic renal failure (serum creatinine had increased from baseline of 160–170 µmol/L to 371 µmol/L). Her admission chest X-ray revealed left lower zone airspace opacities and follow-up computed tomography (CT) of the chest demonstrated bilateral ground glass opacities consistent with pneumonitis (Fig. 1).

**Fig. 2:**
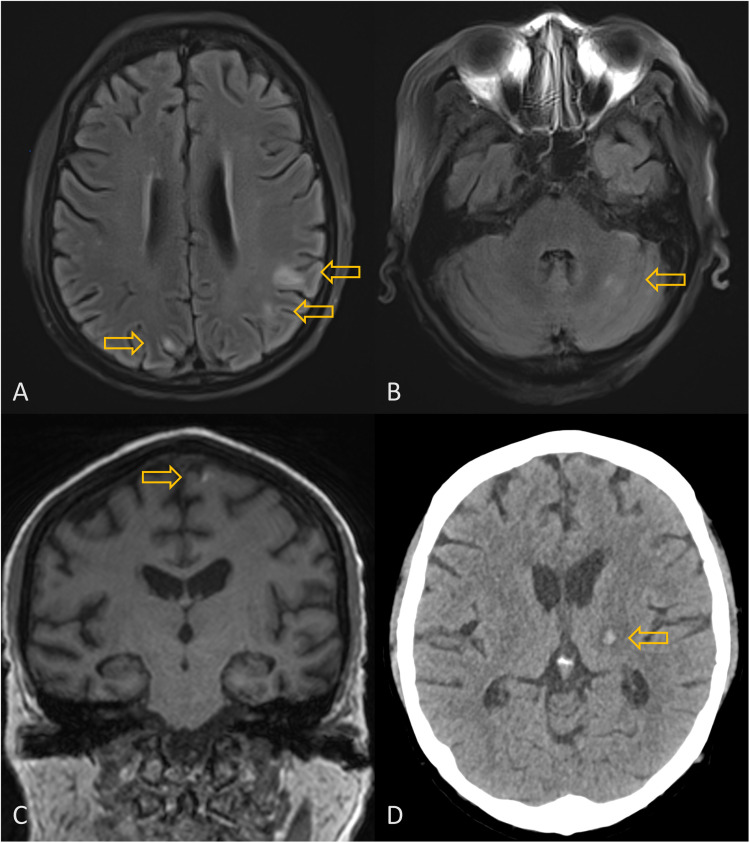
Neuroradiology findings of stem cell transplant recipient with disseminated toxoplasmosis. MRI brain showing multiple scattered FLAIR hyperintense lesions in cerebral (corticomedullary junction) and cerebellar hemispheres (A and B) as well as a left frontal lesion with hyperintense signal on T1-weighted images suggestive of haemorrhage (C). Follow-up CT brain showing a new haemorrhagic lesion at left thalamus (D).

Her admission CT brain was unyielding. In view of renal impairment, a non-contrast enhanced magnetic resonance imaging (MRI) brain was subsequently performed (Fig. 2). Multiple scattered hyperintense lesions were seen on the T2-weighted and fluid attenuated inversion recovery (FLAIR) images, in bilateral cerebral hemispheres (predominantly in the corticomedullary junction in bilateral frontal and parietal lobes with one in the left temporal lobe), left caudate nucleus and bilateral cerebellar hemispheres. A small left frontal lesion showed mild associated hyperintense signal on T1-weighted images and minimal susceptibility on susceptibility weighted imaging (SWI) sequence, suggestive of mild associated haemorrhage. Although the MRI findings were non-specific for toxoplasmosis, the clinical presentation, chest radiological findings were suggestive. Alternative differentials considered at that time included disseminated bacterial / viral infection with meningoencephalitis, other opportunistic infections, PJP (albeit less likely due to the use of pentamidine prophylaxis), and post-transplant lymphoproliferative disease (PTLD). Eventually, polymerase chain reaction (PCR) testing of the serum for Toxoplasma soon returned positive, and her bone marrow aspirate and cerebrospinal fluid also tested positive*,* confirming the diagnosis of disseminated toxoplasmosis. A follow up CT brain showed a new haemorrhagic lesion in the left thalamus (Fig. 2).

Because she initially could not take medications per oral, she was started on intravenous trimethoprim-sulfamethoxazole (TMP-SMX) for 3 days, which was subsequently switched to pyrimethamine and sulfadiazine with leucovorin once a nasogastric tube was inserted. The switch was made due to enteral access becoming possible rather than treatment failure or toxicity. No treatment-limiting adverse effects had been observed. Treatment response was assessed based on clinical improvement and radiographic response. Despite toxoplasma-directed therapy, she continued to have a stormy inpatient stay complicated by recurrent seizures, intracranial haemorrhage, and nosocomial infections. She unfortunately died approximately 1 month into hospitalisation.

## 2 Discussion

Toxoplasmosis is a rare but potentially fatal complication in patients undergoing HSCT. In a systematic review, the prevalence was 399 in 38,751 (1.0 %) HSCT patients. Disseminated infection is even rarer, and was seen in 99 out of 33, 993 (0.3 %) patients after HSCT [2]. To our knowledge, this is the first reported case of disseminated toxoplasmosis following HSCT in Singapore.

Disseminated toxoplasmosis arises as a result of reactivation of latent cysts, beginning with asymptomatic infection (potentially detected by serum polymerase chain reaction (PCR) testing), before progressing to end-organ disease. The organs most commonly affected are the central nervous system (CNS), followed by the lungs [3,4]. Ocular manifestations are rare after HSCT [4,5]. Importantly, the guidelines from the 9th European Conference on Infections in Leukaemia (ECIL-9) recommend routine pre-HSCT serological testing for toxoplasmosis to risk-stratify patients. Prophylaxis or pre-emptive therapy may be considered in the appropriate context [3].

The practice of pre-transplant serologic evaluation, universal screening for toxoplasmosis by PCR after HSCT and initiating pre-emptive therapy had not been adopted in Singapore, largely due to the low background seroprevalence, and thus may not be cost-effective (including testing, follow-up, and therapy), with a lower expected yield. ECIL-9 recommends risk-stratified approaches anchored on local epidemiology; our experience supports reassessing the balance of costs and benefits as new local data emerge In Singapore, the last study on seroprevalence was over 20 years ago; pregnant women, 17.2 % of pregnant women were positive for *Toxoplasma* IgG [6]. These rates are similar in Southeast Asian countries [7]. Comparatively, in France, the reported seroprevalence is 37- 44 %, making testing and pre-emptive therapy for toxoplasmosis more cost-effective [8]. In addition, due to the low seroprevalence of *Toxoplasma*, the condition is often under-diagnosed.

This index case highlights the importance of pre-transplant *Toxoplasma* screening even in settings with low prevalence [9]. In a systematic review, Toxplasmosis was observed in 163 out of 2271 (7.2 %) of seropositive HSCT recipients, compared with 10 out of 6785 (0.1 %) of seronegative HSCT recipients [2]. TMP-SMX prophylaxis for PJP prophylaxis may confer cross-protection. However, breakthrough infections while on prophylaxis are still possible. Toxoplasmosis was reported in as many as 20 out of 117 (17 %) patients while on TMP-SMX prophylaxis [2]. Pentamidine, an alternative for PJP prophylaxis, offers no protection against Toxoplasmosis, and reactivation in seropositive HSCT patients with a compatible clinical syndrome has to be considered. We have now instituted pre-HSCT Toxoplasma serology as part of the pre-transplant workup, and recommend targeted pre-emptive monitoring in seropositive patients, particularly where TMP-SMX prophylaxis is not used.

Timely diagnosis may be challenging. In the appropriate host with a constellation of symptoms that affect both the lungs and CNS, detectable *Toxoplasma* PCR testing of affected tissue such as cerebrospinal fluid or bronchoalveolar lavage may help diagnose probable disease [3,4]. Respiratory involvement may manifest as diffuse alveolar infiltrates, which may resemble PJP [10]. CNS symptoms may be vague and findings on MRI brain may also be non-specific. Most commonly, multiple scattered T2 and FLAIR hyperintense lesions are seen with a predilection for basal ganglia, thalami and corticomedullary junction, although other parts of the brain including brainstem and cerebellum can be involved. Solitary lesions can be encountered, though less commonly, and thus may mimic tumour. Variable surrounding vasogenic edema can be present and some lesions can be haemorrhagic. “Concentric target sign” – alternating concentric zones of hypo/hyper/isointense signal on T2-weighted images has been described as a relatively specific sign for cerebral toxoplasmosis [4]. “Eccentric target sign” – ring enhancement with eccentric enhancing nodule is known as a pathognomonic imaging sign for cerebral toxoplasmosis [4,5]. However, these signs are not always present.

Although the pattern of lesion distribution in the CNS seen in our case is consistent with toxoplasmosis, the findings are non-specific and there is a wide range of differentials. including other opportunistic infections (invasive fungal disease, nocardiosis, viral infections) as well as neoplastic processes such as PTLD. Notably, atypical features have been reported in toxoplasmosis in HSCT patients. They include lack of contrast enhancement for parenchymal lesions [6,7] and presence of leptomeningeal enhancement/disease [1,8]. It has been postulated that the lack of enhancement for parenchymal lesions in toxoplasmosis in HSCT patients compared to patients with acquired immune deficiency syndrome (AIDS) may be related to differences in their immunocompromised state (global loss of immune cells in HSCT versus CD4 cell loss in AIDS) [6].

Prompt initiation of therapy is crucial for improved outcomes in patients with toxoplasmosis. The first-line therapeutic options include pyrimethamine and leucovorin in combination with sulfadiazine or clindamycin or azithromycin, while intravenous TMP-SMX may be an alternative for those who are unable to tolerate per oral [3]. In addition, treatment-related paradoxical worsening of the condition may be expected after initiating appropriate therapy. Particularly for patients with severe CNS or ocular disease, adjunctive corticosteroid therapy may be considered, although no randomised controlled trials exist to support this practice [3]. In patients who survive, secondary prophylaxis is also recommended for those at risk for recurrence (i.e. still remaining of high dose immunosuppression for active graft-versus-host disease, or low CD4 counts) [3].

In conclusion, it may be prudent and beneficial for pre-transplant evaluation of toxoplasma sero-status amongst HSCT recipients in Singapore. This would inform prevention strategies, either primary prophylaxis, or serum PCR testing and pre-emptive treatment for high-risk patients after HSCT, to avert fatal toxoplasmosis.

## Funding/support

None reported.

## Ethics approval and consent to participate

Written informed consent was obtained from the patient prior for this report.

## Consent for publication

All authors consent for the publication of this manuscript.

## Data availability

Data may be made available on reasonable request from the corresponding author.

## CRediT authorship contribution statement

**Jinghao Nicholas Ngiam:** Writing – review & editing, Writing – original draft, Formal analysis, Data curation, Conceptualization. **Thuan Tong Tan:** Writing – review & editing, Formal analysis, Conceptualization. **Ban Hock Tan:** Writing – review & editing, Formal analysis, Conceptualization. **Wenlu Hou:** Writing – review & editing, Formal analysis, Conceptualization. **Aloysius Yew Leng Ho:** Writing – review & editing, Formal analysis, Conceptualization. **Jeffrey Kim Siang Quek:** Writing – review & editing, Formal analysis, Conceptualization. **Yeh Ching Linn:** Writing – review & editing, Formal analysis, Conceptualization. **Francesca Lorraine Wei Inng Lim:** Writing – review & editing, Formal analysis, Conceptualization. **Hein Than:** Writing – review & editing, Formal analysis, Conceptualization. **Shimin Jasmine Chung:** Writing – review & editing, Formal analysis, Data curation, Conceptualization.

## Declaration of competing interest

The authors declare that they have no known competing financial interests or personal relationships that could have appeared to influence the work reported in this paper.

## References

[1] Liew Y.X., Ho A.Y.L., Wong G.C., Chung S.J., Tan T.T., Tan B.H. (2024). A retrospective study of intravenous pentamidine for Pneumocystis jirovecii pneumonia prophylaxis in adult patients with hematologic malignancies—Its utility during respiratory virus pandemics. Int. J. Infect. Diseases.

[2] DG Contopoulos-Ioannidis, Cho S.M., Bertaina A. (2021). Toxoplasmosis among 38 751 hematopoietic stem-cell transplant recipients: a systematic review of disease prevalence and a compilation of imaging and autopsy findings. Transplantation..

[3] Aerts R., Mehra V., Groll A.H. (2024). Guidelines for the management of Toxoplasma gondii infection and disease in patients with haematological malignancies and after haematopoietic stem-cell transplantation: guidelines from the 9th European Conference on Infections in Leukaemia. Lancet Infect. Dis..

[4] Schmidt M., Sonneville R., Schnell D. (2013). Clinical features and outcomes in patients with disseminated toxoplasmosis admitted to intensive care: a multicenter study. Clin. Infect. Dis..

[5] Mele A., Paterson P.J., Prentice H.G., Leoni P., Kibbler C.C. (2002). Toxoplasmosis in bone marrow transplantation: a report of two cases and systematic review of the literature. Bone Marrow. Trans. Plant..

[6] Wong A., Tan K.H., Tee C.S., Yeo G.S. (2000). Seroprevalence of cytomegalovirus, toxoplasma and parvovirus in pregnancy. Singapore Med. J..

[7] Gandahusada S. (1991). Study on the prevalence of toxoplasmosis in Indonesia: a review. Southeast. Asian J. Trop. Med. Public Health.

[8] Nogareda F., Le Strat Y., Villena I., De Valk H., Goulet V. (2014). Incidence and prevalence of toxoplasma gondii infection in women in France, 1980-2020: model-based estimation. Epidemiol. Infect..

[9] Derouin F., Pelloux H. (2008). ESCMID Study Group on Clinical Parasitology. Prevention of toxoplasmosis in transplant patients. Clin. Microbiol. Infect..

[10] de Souza Giassi K., Costa A.N., Apanavicius A. (2014). Tomographic findings of acute pulmonary toxoplasmosis in immunocompetent patients. BMC. Pulm. Med..

